# Exploring the Relationship Between Zinc Status and Frailty: Observational and Genetic Analyses

**DOI:** 10.1093/geroni/igaf122.3587

**Published:** 2025-12-31

**Authors:** Sangyong Choi, Kelin Zhong, Chia-Ling Kuo

**Affiliations:** University of Connecticut, Storrs, Connecticut, United States; University of Connecticut, Storrs, Connecticut, United States; UCONN HEALTH, Farmington, Connecticut, United States

## Abstract

Frailty increases the risk of adverse outcomes in older adults, reflecting diminished physiological reserve and vulnerability to stressors. Zinc, an essential mineral, supports immune function and oxidative stress regulation, with circulating levels generally lower in older than younger adults. This study investigates the potential causal relationship between zinc status and frailty using observational analyses and Mendelian Randomization (MR) in the UK Biobank. Dietary zinc intake was assessed by self-reported questionnaire and categorized per UK guidelines. We assessed frailty in older adults using two complementary indices: the Fried frailty phenotype (physical frailty, defined by ≥ 2 of 5 specific criteria) and a 49-item Frailty Index (FI-49, representing the accumulation of health deficits across multiple systems). Observational analyses revealed divergent associations: excessive zinc intake was linked to higher risk of frailty by FI-49, while zinc deficiency intake was associated with greater risk according to the Fried frailty phenotype, reflecting the conceptual distinctions of each index. MR analyses did not show evidence of a causal effect of genetically predicted blood zinc levels on frailty. These results suggest that both zinc deficiency and excess may contribute to frailty risk, depending on the aspect of frailty measured, while genetically determined zinc levels do not influence status. Despite the possibility of confounding or threshold effects in observational findings, the lack of blood zinc measurements limits conclusions about the relationship between intake, circulating levels, and frailty. Further research should clarify zinc’s effects and assess supplementation in those with low intake or at-risk genotypes.

